# Renal Glomerular Mitochondria Function in Salt-Sensitive Hypertension

**DOI:** 10.3389/fphys.2019.01588

**Published:** 2020-02-04

**Authors:** Mark Domondon, Iuliia Polina, Anna B. Nikiforova, Regina F. Sultanova, Claudia Kruger, Valeriia Y. Vasileva, Mikhail V. Fomin, Gyda C. Beeson, Anna-Liisa Nieminen, Nancy Smythe, Eduardo N. Maldonado, Krisztian Stadler, Daria V. Ilatovskaya

**Affiliations:** ^1^Department of Medicine, Division of Nephrology, Medical University of South Carolina, Charleston, SC, United States; ^2^Institute of Theoretical and Experimental Biophysics, Pushchino, Russia; ^3^Saint-Petersburg State Chemical Pharmaceutical University, Saint Petersburg, Russia; ^4^Oxidative Stress and Disease Laboratory, Pennington Biomedical Research Center, Baton Rouge, LA, United States; ^5^Institute of Cytology Russian Academy of Science, Saint Petersburg, Russia; ^6^Department of Drug Discovery & Biomedical Sciences, Medical University of South Carolina, Charleston, SC, United States; ^7^Department of Pathology, Medical University of South Carolina, Charleston, SC, United States

**Keywords:** kidney, glomeruli, hypertension, podocyte, mitochondria

## Abstract

Salt-sensitive (SS) hypertension is accompanied with an early onset of proteinuria, which results from the loss of glomerular podocytes. Here, we hypothesized that glomerular damage in the SS hypertension occurs in part due to mitochondria dysfunction, and we used a unique model of freshly isolated glomeruli to test this hypothesis. In order to mimic SS hypertension, we used Dahl SS rats, an established animal model. Animals were fed a 0.4% NaCl (normal salt, NS) diet or challenged with a high salt (HS) 4% NaCl diet for 21 days to induce an increase in blood pressure (BP). Similar to previous studies, we found that HS diet caused renal hypertrophy, increased BP, glomerulosclerosis, and renal lesions such as fibrosis and protein casts. We did not observe changes in mitochondrial biogenesis in the renal cortex or isolated glomeruli fractions. However, Seahorse assay performed on freshly isolated glomeruli revealed that basal mitochondrial respiration, maximal respiration, and spare respiratory capacity were lower in the HS compared to the NS group. Using confocal imaging and staining for mitochondrial H_2_O_2_ using mitoPY1, we detected an intensified response to an acute H_2_O_2_ application in the podocytes of the glomeruli isolated from the HS diet fed group. TEM analysis showed that glomerular mitochondria from the HS diet fed group have structural abnormalities (swelling, enlargement, less defined cristae). Therefore, we report that glomerular mitochondria in SS hypertension are functionally and structurally defective, and this impairment could eventually lead to loss of podocytes and proteinuria. Thus, the glomerular–mitochondria axis can be targeted in novel treatment strategies for hypertensive glomerulosclerosis.

## Introduction

Salt-sensitive (SS) hypertension is characterized by elevated blood pressure (BP) resulting from increased dietary salt intake (Pilic et al., 2016; Rust and Ekmekcioglu, 2017). Available medications are insufficient to control BP in the SS subjects, and there is a need for the development of novel effective therapies (Elijovich et al., 2016; Laffer and Elijovich, 2018). To date, substantial research efforts have been devoted to uncovering the mechanisms underlying salt sensitivity, a condition known to be associated with various physiological, environmental, demographic, and genetic factors (Pilic et al., 2016). Intricate studies on kidney transplantations discovered that salt sensitivity “follows” the kidney: SS rats, which received a kidney transplant from a normotensive rat, became salt resistant (Bianchi et al., 1974; Patschan et al., 1997), implying that salt sensitivity originates in the kidney. Glomerular damage, loss of podocytes, and subsequent proteinuria are among the primary signs of kidney disease initiation in SS hypertension, and it is especially compelling to assess the mechanisms underlying their impairment (Denic et al., 2016; Assady et al., 2017; Seccia et al., 2017).

Recent evidence demonstrated that mitochondrial fission/fusion, biogenesis, redox capacity, and homeostasis are implicated in the pathogenesis of hypertensive renal damage, acute kidney injury (AKI), and diabetic nephropathy (DN) (Eirin et al., 2017a; Galvan et al., 2017). Multiple studies (highlighted in recent excellent reviews by Drs. Schnellmann, Sharma, Lerman, Danesh, and other groups) were devoted to identifying mitochondria-related targets for renal dysfunction in CKD. Current knowledge suggests that an imbalance in mitochondrial dynamics and energetics in glomeruli and their components (mesangial cells, endothelial cells, or podocytes), can cause a decrease in ATP production, induction of ROS (reactive oxygen species) generation and the disruption of normal renal function (Zhan et al., 2013; Casalena et al., 2014; Daehn et al., 2014; Ayanga et al., 2016; Long et al., 2016; Szeto et al., 2016; Zhao et al., 2016; Bhargava and Schnellmann, 2017; Duann and Lin, 2017; Eirin et al., 2017a; Galvan et al., 2017; Qi et al., 2017; Sharma, 2017; Sweetwyne et al., 2017; Arif et al., 2019; Kruger et al., 2019; Zhang et al., 2019). However, very little is known about glomeruli and mitochondria in chronic hypertension.

The development of hypertension correlates with changes in mitochondrial dynamics and ROS production in the brain, vasculature, and kidney (Manucha et al., 2015; Dikalov and Dikalova, 2016; Lahera et al., 2017; Loperena and Harrison, 2017; Daneva et al., 2019; Ding et al., 2019). As a major source of ROS, mitochondria can increase sympathetic activity, promoting sodium and volume retention and enhancing vasoconstriction (Dikalov and Dikalova, 2016), although the complete mechanism remains unclear (Loperena and Harrison, 2017). The majority of hypertension-related studies have been focused on mitochondrial function in the tubules. For instance, increased sodium delivery in the medullary thick ascending limb (mTAL) was reported to stimulate mitochondrial H_2_O_2_ production, possibly contributing to SS hypertension (Ohsaki et al., 2012). Urinary mitochondrial DNA (mtDNA) copy number was shown to be elevated in hypertensive patients (Eirin et al., 2016, 2017b). Antioxidant activity of superoxide dismutase 2 (SOD2) attenuated hypertensive effects by scavenging excess renal ROS (Dikalov and Dikalova, 2016). Interestingly, mice deficient in SOD2 develop salt-induced hypertension associated with activation of intrarenal ROS generating pathways (Elliott et al., 1990; Rodriguez-Iturbe et al., 2007). Plenty of evidence support that overproduction of ROS and bioenergetic alterations during hypertension heavily implicate mitochondrial dysfunction and the resulting oxidative stress as key contributors promoting the disease (Manucha et al., 2015; Loperena and Harrison, 2017; Marshall et al., 2018). However, extensive additional mechanistic studies are required to confirm a cause–effect relationship between mitochondrial dysfunction and renal damage (Eirin et al., 2015, 2018).

Cowley et al. (2015) revealed that in the Dahl SS rat there is a natural shift in the redox balance between nitric oxide and ROS, while selective reduction of ROS production in the renal medulla reduces SS hypertension. In another study, they found that the p67(phox) subunit of NADPH oxidase 2 plays a role in the excessive production of renal medullary ROS (Salehpour et al., 2015; Zheleznova et al., 2016). Mitochondrial proteomic analysis and respirometry also revealed deficiencies in oxygen utilization in mTAL and differential expression of mitochondria-encoded proteins in the Dahl SS rat compared to the SS.13(BN) control (Zheleznova et al., 2012). In 2014, He et al. (2014) demonstrated that mitochondrial abnormalities can be observed in the mTAL of SS rats before the development of histologically detectable injury. Later, Wang et al. (2017) provided evidence that renal mitochondria of Dahl SS rats displayed metabolic alterations and dysfunctions, aggravated by an increase in salt intake. Through transcriptomic analyses of renal medullary biological pathways, they established that mitochondrial TCA cycle and cell energetics are crucial for the molecular networks relevant to salt-sensitivity (Cowley et al., 2014; Evans et al., 2018).

Therefore, mitochondrial structure and bioenergetics are critical for the renal mechanisms of SS hypertension development. However, not much is known about renal cortical mitochondria, especially their function in glomeruli. The current study is focused on assessing functional and structural properties of mitochondria in renal glomeruli during SS hypertension. We employed a variety of techniques to address this question in Dahl SS rats, using a preparation of the freshly isolated glomeruli. We hypothesized that glomerular mitochondria function is a central contributor to renal disease development in SS hypertension.

## Materials and Methods

### Study Design and Animal Use

**FIGURE 1:**
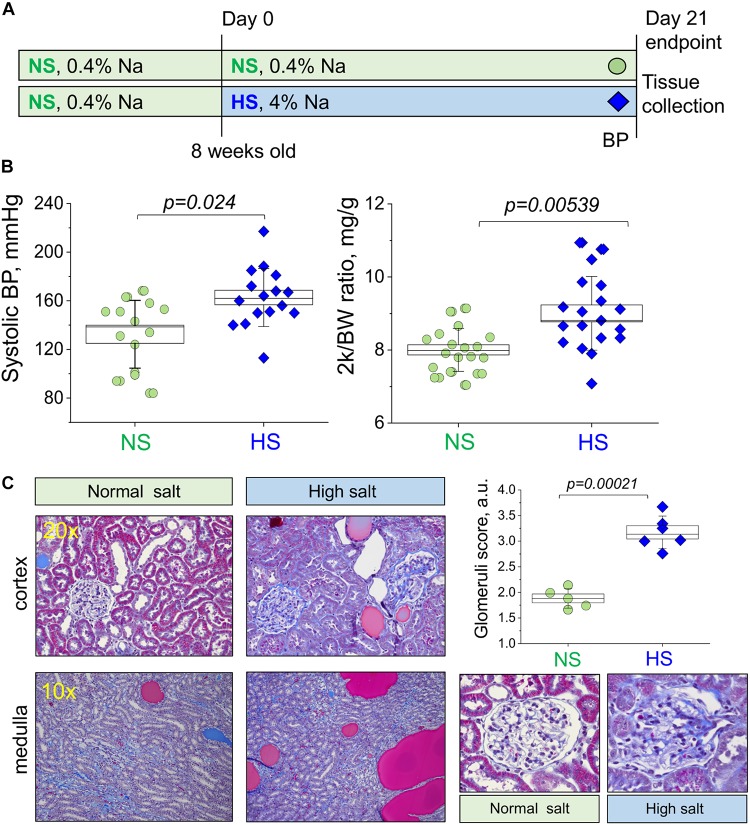
Study protocol and development of the salt-sensitive (SS) hypertension in the Dahl SS rats. **(A)** Schematic representation of the experimental protocol used to induce SS hypertension in Dahl SS rats. **(B)** Graphs illustrating the observed increase in systolic blood pressure (systolic BP, **left**) and two kidney to body weight ratio (middle panel, 2 k/BW) in the NS diet fed group compared to a HS diet fed group (right). **(C)** Representative histological images showing cortical renal tissues (20×) and medullary tissues (10×) from NS and HS diet fed animals stained with Masson’s trichrome. Graph illustrates summarized glomerular damage score (glomeruli examples are provided below for each experimental group). All data were compared using a one-way ANOVA with Holm–Sidak *post hoc* test. In Graphs **(B)**, each data point represents a single measurement from an experimental animal at the end of the protocol after the NS or HS challenges. For the glomerular damage score **(C)**, each point is an average of 100 glomeruli blindly scored in the renal tissue of each animal. NS, normal salt; HS, high salt.

Male Dahl salt-sensitive (Dahl SS) rats were obtained from Charles River Laboratories (strain code 320) at 7 weeks of age, and they were switched to a normal salt (NS, 0.4% NaCl) AIN-76A-based diet upon arrival (Dyets, Inc., #D113755, Bethlehem, PA, United States). At 8 ± 0.3 weeks of age (265 ± 15 g body weight), rats were either maintained on a NS diet or provided a high salt (HS, 4% NaCl) AIN-76A-based chow (Dyets, Inc., D#113756) to induce hypertension development (Figure 1A). Rats were housed at the Medical University of South Carolina (MUSC) Department of Laboratory Animal Resources under 12 h light/dark cycle. Access to food and water and environmental enrichment were provided *ad libitum*. All animal experiments were conducted in accordance with the National Institutes of Health Guide for the Care and Use of Laboratory Animals following the protocol review and approval by the MUSC Institutional Care and Use Committee. Rats were randomly assigned to research groups; sample size was estimated using power analysis. Euthanasia and tissue collections were performed between 10 am and 2 pm.

### Blood Pressure Measurements, Kidney Flush, and Glomeruli Isolation

Blood pressure measurements via tail cuff plethysmography (IITC Life Science Inc., United States) were obtained from each rat at 11 weeks old, immediately before endpoint kidney flush. For tissue collections, rats were anesthetized with 2.5% isoflurane, abdominal aorta was catheterized for blood collection, and kidneys were flushed with PBS (3 ml/min/kidney until blanched) as described previously (Ilatovskaya et al., 2015b). Then, tissues were snap-frozen for Western blotting or qPCR, fixed for subsequent histological or electron microscopy analyses, or used for immediate *ex vivo* experiments. For glomeruli isolation, renal cortex was excised and minced using a single-edged razor blade; then, isolation was performed with differential sieving as described previously (Ilatovskaya et al., 2011, 2015b; Ilatovskaya and Staruschenko, 2013). Briefly, the minced tissue was sequentially pushed through a steel 150-μm sieve and then pipetted through a 106-μm sieve (04-881-5Z and 04-881-5X; Fisher Scientific) using the culture medium solution RPMI1640 (Invitrogen, Inc., United States) with 5% BSA. This tissue homogenate was then pipetted onto a 75-μm sieve (S4145; Sigma), rinsed from the sieve surface, and stored on ice. Glomeruli were used within 3 h post isolation.

### Histological Staining and Glomeruli Scoring

Tissues fixed with 10% NBF were routinely embedded, cut and mounted on slides, deparaffinized, rehydrated, and stained with Masson’s trichrome. Glomeruli scoring was performed according to previously published protocols and scales (Raij et al., 1984; Palygin et al., 2017) using a Nikon Ti-2 microscope equipped with a 40× NA 0.7 objective and a DS-Fi2 color camera; glomeruli were blindly scored from zero (healthy) to four (diseased) (see Raij et al., 1984). At least 100 glomeruli were randomly scored in the cortical area of each experimental animal.

### Plasma Creatinine and Electrolyte Measurements

Blood samples obtained from the abdominal aorta before kidney flushing, were centrifuged immediately after collection at 6000 rpm for 5 min to separate the plasma. The plasma was snap-frozen and stored at −80°C. Plasma creatinine levels were measured using the Quantichrom Creatinine Assay Kit (DICT-500). A standard curve was created from the stock 50 mg/dl creatinine standard. Concentrations of 6, 2, 1, 0.5, and 0 mg/dl were used to create the standard curve. Creatinine concentrations were determined by measuring absorbance per the manufacturer’s instructions. Plasma electrolyte levels were measured with Carelyte analyzer (Diamond Diagnostics, United States).

### Electron Microscopy

Samples were excised from the animal and fixed overnight in freshly made 2.5% glutaraldehyde in phosphate buffer (Electron Microscopy Sciences). The samples were rinsed in buffer 2× for 15 min and postfixed in 2% osmium tetroxide for 1 h on a rocker plate. Each sample was then dehydrated through a series of ethyl alcohol dilutions starting at 50, 70, 90, and 95%. Three 100% rinses for 15 min were used to complete the dehydration, and the samples were put into propylene oxide to start the infiltration with Embed 812 (Electron Microscopy Sciences) at ratios of 1:3, 2:2, and 3:1 for 1 h each. In the final stage the samples were then left in pure plastic overnight on the rocker plate and subsequently put into the mold and left in the oven overnight to polymerize. Once hardened, the blocks were trimmed, semi-thick sectioned at 0.5 μm, dried on a glass slide, stained with 1% toluidine Blue, and looked at under the microscope to determine the appropriate area to thin section. The block was trimmed again to reflect that area, and the thin sections were taken and placed on a 200-mesh cu grid, dried and stained with uranyl acetate for 10 min, and rinsed and stained with lead citrate for 10 min. The sections were observed on a JEOL 1010 transmission electron microscope running at 80 kV and imaged.

### Protein and mRNA Isolation, Western Blotting, and Mitochondrial DNA Quantification

After excision, kidneys were cut into 1–2 mm slices, and the cortical kidney pieces were pulse sonicated in RIPA buffer containing a protease inhibitor cocktail (Roche) on ice for up to 10 s and then spin cleared at 10,000 × *g* for 10 min. The resulting supernatant was subjected to PAGE, transferred onto a nitrocellulose membrane (Bio-Rad, Hercules, CA, United States) for probing with antibodies, and subsequently visualized by enhanced chemiluminescence (ECL; Thermo Scientific, Waltham, MA, United States). The following antibodies were used: Total OXPHOS Rodent WB Antibody cocktail (ab110413, abcam, 1:500), PGC1α Polyclonal Antibody (PA5-38022, Invitrogen, Inc., United States); secondary anti-Mouse IgG HRP Conjugate (W402B, Promega, 1:5,000), and Goat anti-Rabbit IgG (H + L) (#31460, Invitrogen, Inc., United States). For qPCR, total DNA was extracted from rat kidney cortical tissues using a DNeasy Blood and Tissue kit (69504; Qiagen). The quality of each sample was determined using gel electrophoresis of 50 ng of total DNA following PCR on a C1000 Themocycler (Bio-Rad, Hercules, CA, United States) with a SuperScript First-Strand synthesis system for RT-PCR (11904; Invitrogen, Inc., United States), while the quantity was determined by spectrophotometry (Nanodrop). Real-time PCR analysis was performed with 50 ng total DNA using SYBR green chemistry on a CFX96 (Bio-Rad, Hercules, CA, United States). Primer sequences were as follows: *Nd1* F: ATGGCCTTCCTCACCCTAGT, R: GTTAGGGGGCGTATGGGTTC, *Nd6* F: TTGGGGTTGCGG CTATTTAT, R: ATCCCCGCAAACAATGACCA, with positive controls *B-actin* F: GCTCTATCACTGGGCATTGG and R: CGCAACTCTTAACTCGGAAGA.

### Confocal Microscopy and Image Analysis

Isolated glomeruli were allowed to adhere onto glass bottom Mat-Tek dishes coated with poly-L-lysine for 5 min (P4707; Sigma) and immediately imaged in a solution containing (in mM): 145 NaCl, 4.5 KCl, 2 CaCl_2_, 2 MgCl_2_, 10 HEPES, pH 7.35. Fluorescence intensities were monitored using a confocal laser scanning microscope system Leica TCS SP 5. Detection was performed using an oil immersed 63×/NA 1.4 Oil objective with the argon laser excitation at 488 or 361 nm and emission filters 520/25 nm for MitoPY1 (Tocris, #4428, 10 μM loading for 30 min at RT) and 480/25 nm for Hoechst 33342 (ThermoFisher, H3570, 8 μM loading for 10 min at RT), respectively. Fluorescence images were collected with 0.125 Hz frequency and processed with open source software Fiji (ImageJ 1.47v, National Institute of Health, United States). Podocytes were located on the surface of capillaries by their morphology, body size, nuclei size, and structure in the focal plane next to the glass chip, similarly to what was done in previously published manuscripts (Ilatovskaya and Staruschenko, 2013; Ilatovskaya et al., 2015a, b, 2018). Please see Figure 5A for a detailed schematic.

### Complex V Activity Assay

Mitochondrial Complex V activity was analyzed in isolated glomeruli pellets, normalized to 10 μg protein, and expressed as oxidation rate of NADH at 340 nm. The reaction mixture in a 1 cm cuvette contained: 500 μl of Buffer HEPES-Mg, 10 μl of 30 mM NADH, 50 μl of 50 mM phosphoenolpyruvic acid, 5 μl of 10 mg/ml of pyruvate kinase, 10 μl of 5 mg/ml of lactate dehydrogenase, and 10 μl of antimycin A. Ten micrograms of protein was added, and the mixture was incubated at 30°C for 2 min. The baseline kinetics were then recorded for 2 min. The reaction was started by adding 100 μl of 25 mM ATP, and kinetics were measured for 2 min (ΔAbs 340). Then 10 μl oligomycin (0.2 mg/ml in 50% ethanol) was added, and absorbance was measured again for 2 min. Complex V specific activity was calculated by comparing the Δabs with and without oligomycin.

### Oxygen Consumption Rate Measurements

Equal volumes of freshly isolated glomeruli were plated onto Agilent Seahorse XF96 Microplates (Agilent Technologies, CA, United States, #101085-004). The Seahorse XF96 Analyzer (Agilent Technologies, CA, United States) was used to measure the basal levels of oxygen consumption rate (OCR) and OCR in the presence of electron transport chain (ETC) inhibitors and uncouplers (oligomycin, 1 μM; carbonyl cyanide-*p*-trifluoromethoxyphenylhydrazone, 1 μM; rotenone, 2 μM; antimycin A, 1 μM) using the standard software algorithm. The day before analysis, the sensor cartridge was placed in the calibration buffer provided by Seahorse Agilent. The following day, the medium was replaced with low phosphate DMEM buffer and warmed up in a 37°C non-CO_2_ incubator. The injection ports of the sensor plate were filled with 25 μl of the compounds or vehicle diluted in buffer. The sensor plate was placed into the XF-96 instrument for calibration. After calibration, the calibration fluid plate was removed, and the cell plate was loaded for analysis. The measurement protocol was 2 min mix and 3 min measurement. There were three rate measurements post each injection (basal levels, oligomycin, FCCP, and antimycin/rotenone), and each injection had three measurement cycles. OCR was finally normalized to protein concentrations (a standard BCA assay was performed on tested wells and OCR results were normalized to protein content).

### MitoSox Fluorescence Assay

Isolated glomeruli were incubated with 5 μM MitoSOX (M36008, Invitrogen, Inc., United States) for 25 min at room temperature with continuous mixing on a rotating shaker. Then, MitoSOX fluorescence (510-nm excitation/580-nm emission) was measured in multiple 96-well plates using a fluorescence plate reader (Novostar, BMG LabTechnologies, Offenburg, Germany). Fluorescence was normalized to 1 mg of protein.

### Antioxidant Kit

Antioxidant Assay (Cayman Chemical, Item No. 709001) was used to measure the total antioxidant capacity of kidney cell lysates. Kidney tissue (∼10–15 mg/sample) from each studied group was sonicated in the assay buffer on ice, then cells were spinned down by centrifugation (10,000 × *g* for 15 min at 4°C). The supernatant was diluted 1:50 and used for further analysis according to the manufacturer’s instructions. Absorbance was measured in 96-well plates with a Biotek Instruments plate reader at 405 nm. The capacity of the antioxidant system was compared with a standard curve obtained with Trolox (water-soluble tocopherol analog) and quantified as milliMolar (mM) Trolox equivalents.

### RNA Isolation

RNA from rat glomeruli was isolated using TRIzol Reagent (Life Technologies) according to the manufacturer’s instructions. In short, tubes containing glomeruli in TRIzol were thawed at room temperature. Fifty microliters of chloroform was added, and the contents were mixed by vortexing for 10 s before incubating at room temperature for 5 min. After centrifugation at 15,300 × *g* for 15 min at 4°C, the aqueous layer was carefully transferred to a new set of tubes and 1 μl glycogen (Thermo Fisher Scientific), 100 μl isopropanol (Fisher Scientific), and 10 μl 8 M LiCl (Sigma-Aldrich) were added to assist precipitation at −20°C overnight. The next morning centrifugation was performed at 21,000 × *g* for 40 min at 4°C, and the pellet was washed using 70% ethanol. After centrifugation at 15,300 × *g* for 30 min at 4°C all ethanol was removed, and the pellet was allowed to air dry. Then, the RNA pellet was dissolved in 20 μl nuclease-free water and incubated at 60°C for 15 min. RNA integrity was measured using the RNA 6000 Nano chip on the Agilent 2100 Bioanalyzer (Agilent Technologies, CA, United States). Acquired RNA integrity numbers (RIN) were >5.8 for all investigated samples.

### Reverse Transcription

The High-Capacity cDNA Reverse Transcription Kit with RNase inhibitor (Applied Biosystems) was used to reverse transcribe 800 ng RNA from each sample into cDNA. In brief, the 20 μl reaction consists of 10 μl 2× RT Master Mix and 10 μl RNA sample. The Master Mix contains 2.0 μl 10× RT Buffer, 0.8 μl 25× dNTP Mix (100 mM), 2.0 μl 10× RT Random Primers, 1 μl MultiScribe Reverse Transcriptase, 1 μl RNase Inhibitor, and 3.2 μl Nuclease-free water. The thermal cycling conditions were as follows: 10 min at 25°C, 120 min at 37°C, 5 min at 85 and 4°C. After cycling, synthesized cDNA was transferred into a new tube and 60 μl nuclease-free water was added to a final concentration of 10 ng/μl for each sample.

### Primers

Sequences of the target genes were downloaded from the Ensembl genome browser. Primer Express Software v3.0 (Applied Biosystems) was used in its default settings for primer pair design. Where possible, the amplicon was spanned across an exon–exon boundary to exclude potential contamination with genomic DNA. The locations and sequences of the primers are listed in Table 2. Synthesis of the primer sets was carried out by Integrated DNA Technologies (Coralville, IA, United States). Primers were reconstituted with low-TE (Thermo Fisher) to a concentration of 100 μM and diluted with nuclease-free water (Ambion) to a working solution with 10 μM concentration.

### Quantitative Real-Time PCR

Quantitative Real-Time PCR was performed on the 7900HT Sequence Detection System (Applied Biosystem) with default settings including dissociation curves for each assay. Using the epMotion 5075 (Liquid Handling Robot from Eppendorf), the PCR reactions were set up in a 384-well plate (Applied Biosystems) with 2 ng cDNA per reaction. Total volume was 10 μl per reaction [consisting of 3 μl cDNA, 5 μl iTaq Universal SYBR Green Supermix containing ROX as passive reference dye (Bio Rad), 1.8 μl water, 0.1 μl forward primer, and 0.1 μl reverse primer]. The thermal cycling reaction was started with 2 min at 50°C and 10 min at 95°C for optimal DNA polymerase activation. The PCR reactions consisted of a denaturation step of 15 s at 95°C, annealing and extension for 1 min at 60°C, for a total of 40 cycles. Reactions were run in triplicates, including no-template controls (water) for each gene. The comparative C_T_ method (2^–ΔΔ*CT*^) was used for relative quantification of gene expression (User Bulletin #2, Applied Biosystems). For each primer set, the actual amplification efficiency (AE) was calculated and implemented in the formula to calculate fold-changes. The geometric means of measurements for peptidylprolyl isomerase B (Ppib) and Sod2 gene expression were used as the references for normalization to derive Δ*C*_t_ values. For each primer pair, the AE was calculated by applying the formula: ΔRn_cycle (n)_/ΔRn_cycle (n–1)_ over three consecutive cycles, starting at the determined *C*_t_ value in the geometric phase.

### Statistical Analysis

All data are displayed as box plots showing all data points. The box represents SEM, the error bars show SD, and a horizontal line denotes the median value. One-way ANOVA with Holm–Sidak *post hoc* or one-way repeated-measures ANOVA with Holm–Bonferroni for *post hoc* means comparison was employed for statistical analysis; *p*-value <0.05 was considered statistically significant. Animal numbers in each group and the number of replicates, if applicable, as well as the statistical tests applied, are shown in the corresponding figure legends. Statistical analysis of comparisons between conditions in qPCR experiments was conducted using *t*-tests, assuming unequal variances and two-sided *p*-values. *P*-values <0.05 were considered significant. DataAssist software (Applied Biosystems) was used to identify possible outliers among triplicate measurements.

## Results

### Hypertension and Renal Injury Development in Dahl SS Rats

Male Dahl SS rats (Charles River Laboratories) were placed on a 0.4% NaCl diet immediately after arrival. Animals were randomly assigned to NS and HS diet fed groups following the standard experimental protocol (Figure 1A). After 21 days on a HS diet, rats developed a typical hypertension. Systolic BP as measured with tail cuff technique was 132.4 ± 7.4 and 162.7 ± 5.9 mmHg in the NS and HS diet fed groups, respectively (Figure 1B). Two kidneys to body weight ratio was increased in the HS diet fed group (Figure 1B, right panel, 8.0 ± 0.1 vs. 9.0 ± 0.2 mg/g in the NS diet fed group). Additional physiological parameters measured at the end of the experimental protocol (plasma creatinine, electrolyte levels, heart weight, and body weight, reported in Table 1) were similar. To characterize the renal lesions in the experimental groups, we performed blinded glomerular damage score in the Masson’s trichrome stained tissues. As shown in Figure 1C, there was an increase in renal protein cast formation in the HS diet fed animals as well as pronounced glomerular damage reflected in the overall enlarged glomeruli, reduced size of Bowman space, blocked capillaries, mesangial matrix expansion, loss of podocytes, and fibrosis. The glomerular damage score was 1.8 ± 0.1 vs. 3.2 ± 0.1 in the NS and HS diet fed groups, respectively. Therefore, the well-developed renal injury and BP increase in our model were in accordance with previously published studies (Endres et al., 2017; Palygin et al., 2017; Pavlov et al., 2017; Ilatovskaya et al., 2018; Kumar et al., 2019).

**TABLE 1:** Various physiological parameters measured at the end of the experimental protocol in the high salt (HS) and normal salt (NS) diet fed groups.

	NS diet	HS diet	*p*-value	*N*
Plasma [Na], mM/L	148.3 ± 6.0	146.4 ± 2.5	0.32	12
Plasma [K], mM/L	3.8 ± 0.4	3.9 ± 0.4	0.54	12
Plasma [Cl], mM/L	105.5 ± 3.2	107.7 ± 2.1	0.06	12
Plasma creatinine, mg/dl	0.93 ± 0.2	0.83 ± 0.1	0.24	6
Body weight, g	343.8 ± 16.9	348.5 ± 16.4	0.46	14
Heart weight, g	1.3 ± 0.1	1.3 ± 0.1	0.75	14

*At least six animals per group, data are shown ± SEM.*

**TABLE 2:** Genes investigated in this study: primer location, sequences, and amplification efficiency.

Gene symbol	Ensembl ID	Gene name	Forward primer; sequence	Forward primer position
Atp5f1a	ENSRNOG00000017032	ATP synthase F1 subunit alpha	TGACCGAGCTGCTAAAGCAA	1,449–1,468
Cox4i1	ENSRNOG00000017817	Cytochrome c oxidase subunit 4i1	CCATGTTCTTCATCGGCTTCA	417–437
Cycs	LOC100363502-201	Cytochrome c, somatic-like	GGCTGCTGGATTCTCTTACACA	204–225
Gpx4	ENSRNOG00000013604	Glutathione peroxidase 4	AGGCAGGAGCCAGGAAGTAATC	496–517
Ppargc1a	ENSRNOG00000004473	PPARG coactivator 1 alpha	AAGGTCCCCAGGCAGTAGATC	1,906–1,926
Ppib	ENSRNOG00000016781	Peptidylprolyl isomerase B	GGCTCCCAGTTCTTCATAACTACAG	561–585
Sod2	ENSRNOG00000019048	Superoxide dismutase 2	TGCCGCCTGCTCTAATCAG	566–584

**Gene symbol**	**Reverse primer; sequence**	**Reverse primer position**	**Exon–exon boundary**	**AE**

Atp5f1a	TGACAGCCACCTGTTCTTCAAT	1,511–1,490	Yes	1.96
Cox4i1	GGGCCATACACGTAGCTCTTCT	480–459	Yes	1.91
Cycs	TTTCCAAATACTCCATCAGGGTATC	286–262	Yes	1.88
Gpx4	CAGATCTTGCTGTACATGTCAAACC	578–554	Yes	1.90
Ppargc1a	GGTGTCTGTAGTGGCTTGATTCAT	1,973–1,950	Yes	1.89
Ppib	ACCACATCCATGCCTTCCA	652–634	Yes	1.95
Sod2	CGTGCTCCCACACATCAATC	645–626	Yes	1.91

*Gene sequences were taken from the Ensembl genome browser, and the positions corresponding to primer sequences (in 5′–3′ direction) are listed. Primer pairs were designed to span across an exon–exon boundary. The achieved amplification rate over three cycles in the geometric phase with each primer set is listed.*

### Dietary Salt Challenge Does Not Affect Mitochondrial Biogenesis and the Expression of ETC Complexes in Renal Cortex and Isolated Glomeruli

**FIGURE 2:**
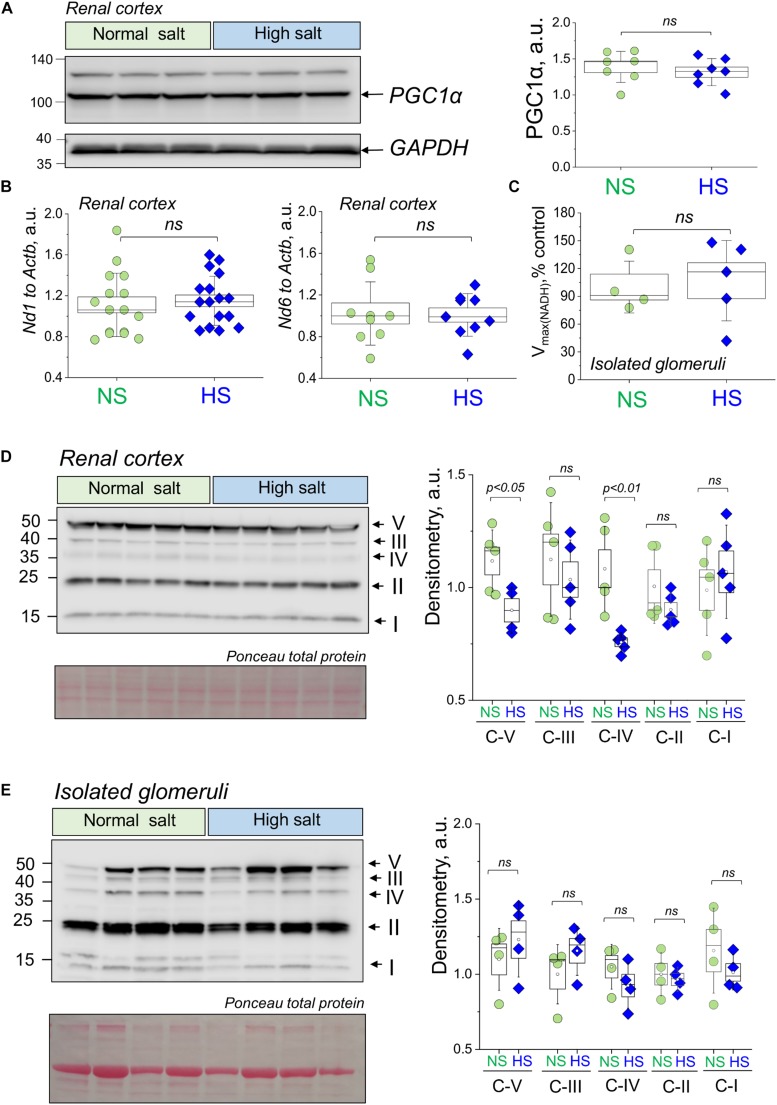
Renal mitochondria biogenesis and electron transport chain (ETC) complexes expression and activity during salt-sensitive (SS) hypertension. **(A)** Representative Western blot showing expression of PGC1α in the renal cortex of the HS and NS diet fed animals. Graph on the right summarizes densitometry values from the Western blot shown on the left combined with another (independent) Western run on other samples (data not shown). All data were normalized to GAPDH expression level. *N* = 7 individual rats tested in each group. **(B)** Mitochondrial DNA content in the renal cortex of rats fed a HS or a NS diet. Shown are results of qPCR for mitochondria genome encoded genes *Nd1* and *Nd6* (values normalized to nuclei-encoded *Actb*). **(C)** ETC Complex V activity as measured in the glomeruli isolated from NS and HS diet fed rats. Data were normalized to protein level, *n* = 5 animals per group. **(D,E)** Western blot analysis illustrating the expression of ETC complexes V, IV, III, II, and I in the renal cortex **(D)** and isolated glomeruli **(E)** from HS and NS diet fed Dahl SS rats. Graph on the right summarizes densitometry values (normalized to total protein as measured with Ponceau stain). NS, normal salt; HS, high salt; ns, not statistically significant (*p* > 0.05). All data were compared using a one-way ANOVA with Holm–Sidak *post hoc* test. Each lane on the Western is a sample from a separate animal.

**FIGURE 3:**
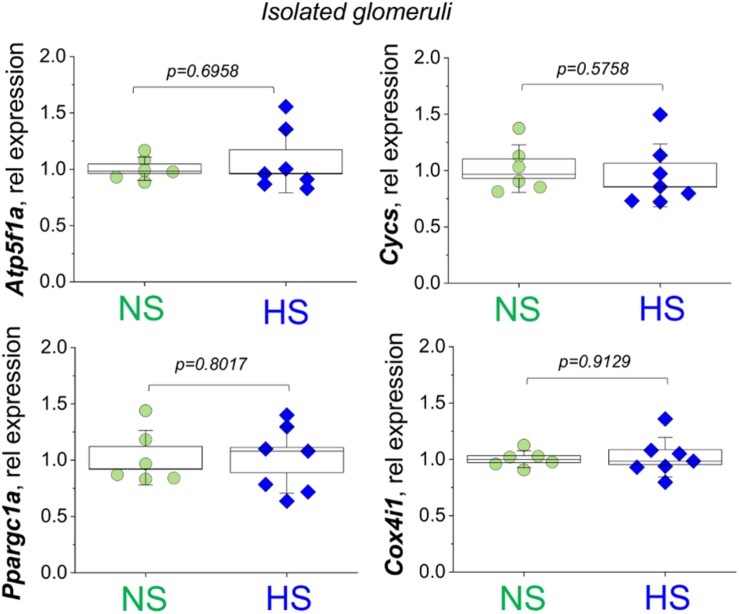
Relative expression of the *Atp5f1a*, *Cycs*, *Ppargc1a*, and *Cox4i1* genes assessed in the fraction of the glomeruli isolated from normal salt (NS) and high salt (HS) diet fed Dahl SS rats. Each point on the graph is a sample from a separate animal. *P*-values were obtained with *t*-tests, assuming unequal variances.

We first aimed to test if renal cortical mitochondria biogenesis was affected by a HS challenge. As reported in Figure 2A, the expression of PGC1α protein (Peroxisome Proliferator-Activated Receptor-Gamma Coactivator-1α), a transcriptional coactivator that regulates the genes involved in energy metabolism and a master regulator of mitochondrial biogenesis, was similar between groups. We further confirmed this finding by testing for another mitochondrial biogenesis factor (Gibbs et al., 2018; Cameron et al., 2019) – the mitochondria DNA content (mtDNA copy number) in the renal cortex by qPCR. As demonstrated in Figure 2B, this experiment did not reveal differences in the mitochondria-encoded *Nd1* and *Nd6* gene expression (normalized to genomic DNA encoded *Actb*). Next, we assessed the mitochondrial Complex V (the ATP synthase of the mitochondrial ETC) activity in isolated glomerular pellets (expressed as oxidation rate of NADH at 340 nm). As shown in Figure 2C, we did not report any significant changes in this parameter between the groups, which is supportive of the data reported above regarding similar biogenesis and mtDNA content in the HS and NS diet fed rats. We next tested the expression of the mitochondrial oxidative phosphorylation (OXPHOS) complexes in the renal cortex and glomeruli using a well-established cocktail of antibodies available from Abcam. We reported a slight decrease in Complex V (ATP synthase) and Complex IV (Cytochrome c oxidase) expression (Figure 2D), whereas other OXPHOS proteins [CIII (Q-cytochrome c oxidoreductase), CII (succinate-Q oxidoreductase), CI (NADH-coenzyme Q oxidoreductase)] were not affected. We further tested if OXPHOS expression changes in isolated glomeruli fraction (Figure 2E), and densitometry analysis showed that the expression of Complexes I, II, III, IV, and V was similar between groups. In addition, we supported these data with qPCR performed in the freshly isolated glomeruli (Figure 3): expression analysis of *Ppargc1a* (encoding for PGC1α), *Cycs* (encoding for Cytochrome C), *Atp5f1a* (encoding for mitochondrial ATP synthase, complex V), and *Cox4i1* (encoding for Cytochrome C Oxidase Subunit 4I1) did not reveal any differences in glomeruli isolated from the NS and HS diet fed rats.

### Glomerular Mitochondrial Respiration Is Suppressed in Glomeruli Isolated From Rats Fed a HS Diet

**FIGURE 4:**
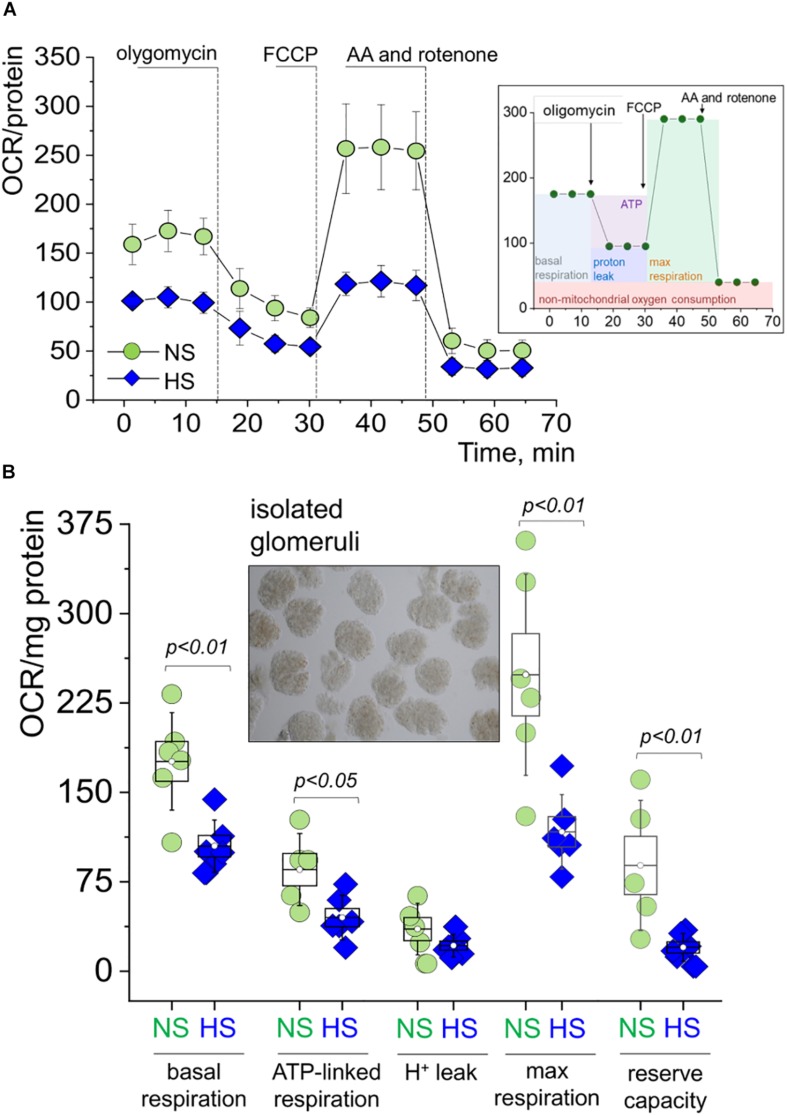
Mitochondrial respiration in the freshly isolated glomeruli during SS hypertension. **(A)** Oxygen consumption rate (OCR, pmol/min/mg protein) values obtained in a Seahorse experiment from glomeruli freshly isolated from the renal cortex of rats fed a high salt (HS) or normal salt (NS) diet. Shown are curves summarized from at least five rats per group, three to five replicates per rat; error bars are SEM. Right inset panel illustrates the experimental design. **(B)** Summary for basal respiration, ATP production, proton leak, maximal respiration, and reserve respiratory capacity as measured in the seahorse experiment shown in Graph. OCR is expressed in pmol/min/mg protein **(A)**. Each data point is an average of at least three replicates per individual rat. An inset shows a typical preparation of isolated glomeruli. All data were compared using a one-way ANOVA with Holm–Sidak *post hoc* test. NS, normal salt; HS, high salt.

Freshly isolated decapsulated glomeruli were used in the OCR experiments on an Agilent Seahorse assay, which is a standard procedure for analytical detection of mitochondrial respiration. Cellular oxygen consumption (respiration) causes changes to the concentrations of oxygen dissolved in the media, measured by solid state sensor probes. Different states of respiration are assessed in a so-called “mitochondrial stress test,” where ETC proteins are targeted with specific inhibitors. In this standard assay, we added oligomycin [blocks the proton channel of ATP synthase (V), so phosphorylation of ADP is no longer possible], FCCP (uncoupler of OXPHOS, which allows the ETC to function at the maximal respiratory capacity), as well as antimycin and rotenone (complex III complex I inhibitors, which shut down the entire ETC). A schematic illustration of the experimental protocol is shown on an inset in Figure 4A. As seen from the summarized OCR transients in Figure 4A and dot plots derived from these curves (Figure 4B), glomeruli from the HS fed rats had significantly impaired respiration. Specifically, we observed higher basal respiration (oxygen consumption used to meet cellular ATP demand under baseline conditions, 176.0 ± 16.7 vs. 104.9 ± 8.9 OCR/mg protein) and ATP-linked respiration (the portion of basal respiration that was being used to drive ATP production, 85.3 ± 12.3 vs. 45.0 ± 8.9 OCR/mg protein) in the NS group vs. the HS groups. Both maximal respiration (FCCP stimulates the respiratory chain to operate at maximum capacity and causes rapid oxidation of substrates to meet the metabolic challenge: 248.7 ± 34.4 vs. 117.0 ± 12.7 OCR/mg protein) and spare respiratory capacity (difference between maximal and basal respiration, 88.7 ± 22.3 vs. 20.0 ± 4.8 OCR/mg protein) were higher in the NS group vs. the HS group, respectively. All data were normalized to protein content. Proton leak (remaining basal respiration not coupled to ATP production) was not found to be significantly different between the groups.

### Glomerular Mitochondria From HS Diet Fed Rats Reveal Structural and Functional Changes as Well as Mitochondria-Derived Oxidative Stress

**FIGURE 5:**
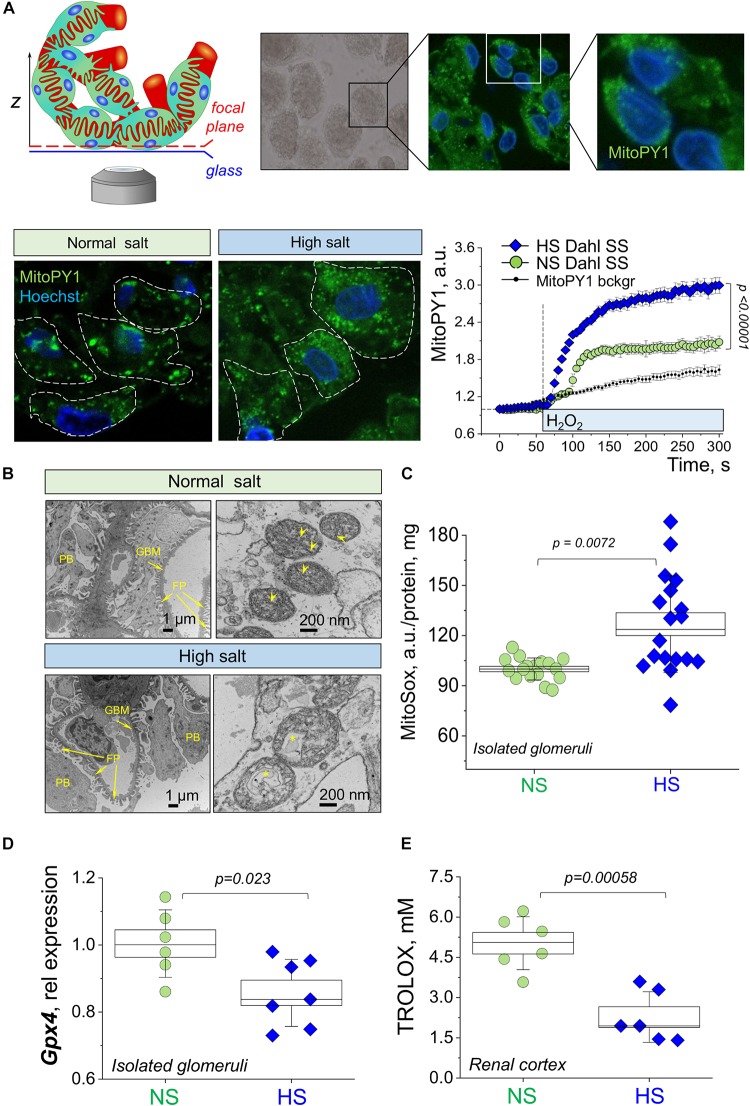
Structural and functional properties of glomerular mitochondria in salt-sensitive (SS) hypertension. **(A)** A schematic illustration of the imaging configuration, as well as representative images of the podocytes in the isolated glomeruli stained with mitoPY1 to visualize H_2_O_2_ in mitochondria (green), and Hoechst (blue) to label nuclei. Images were obtained with a 63× oil objective, NA 1.4. Dotted lines show podocyte body outlines (obtained from TL images). Graph on the right summarizes the fluorescence transients obtained from mitoPY1-stained podocytes of freshly isolated glomeruli in response to acute addition of 10 μM H_2_O_2_. Background auto fluorescence of mitoPY1 is shown (transient obtained in the absence of H_2_O_2_). *N*, five rats per group, at least four glomeruli per animal, with five to nine ROIs (podocytes) analyzed per glomerulus. Statistical analysis performed using one-way repeated measures ANOVA with Holm–Bonferroni for *post hoc* means comparison. **(B)** Representative electron microscopy images of the mitochondria ultrastructure in the podocyte of the glomeruli isolated from NS and HS diet fed rats. Scale bar is shown on the graph; asterisk denotes autophagosome formation; arrowheads show cristae. FP, foot processes; GMB, glomerular basement membrane; PB, podocyte body. **(C)** Mitochondria of glomeruli of HS diet fed animals exhibit higher levels of super oxide, as shown by fluorescence of MitoSox, six rats in each group, three replicates were measured per each experimental animal. Data were compared using a one-way ANOVA with Holm–Sidak *post hoc* test. **(D)** Relative expression of *Gpx4* in the fraction of freshly isolated glomeruli. **(E)** The antioxidant capacity of renal cortical tissue compared in NS vs. HS diet fed animals (expressed in mM Trolox). Data were compared using a one-way ANOVA with Holm–Sidak *post hoc* test, each point represents a separate animal.

In order to examine mitochondrial function in renal glomeruli in further detail, we performed confocal imaging of podocytes from freshly isolated glomeruli. These samples were loaded with a MitoPY1 dye to label mitochondria-produced hydrogen peroxide. Figure 5A demonstrates a schematic of glomeruli imaging and representative staining of the podocytes in the glomerular sample [the technique for labeling and imaging podocytes in freshly isolated glomeruli was well established in our earlier publications (Ilatovskaya et al., 2015a, b, 2018; Spires et al., 2018)]. These experiments revealed a more fragmented staining pattern for mitochondrial H_2_O_2_ in the glomeruli from the HS diet fed rats. We believe that the observed staining pattern indicates the enhanced H_2_O_2_ formation and is reflective of the ultrastructural changes in the mitochondria that are being observed with EM (formation of the mitophagosomes, which could result in staining artifacts, such as fragmentation). In addition, we assessed an acute effect of H_2_O_2_ on mitoPY1-stained glomeruli, and observed a significantly blunted response in the NS diet fed glomeruli, indicative of higher antioxidant capacity in this tissue. Next, isolated glomeruli TEM demonstrated a pronounced structural damage to the mitochondria in the podocytes from the HS diet fed animals (as shown by loss of cristae, swelling, and signs of mitophagy) compared to the NS glomeruli as seen in representative images in Figure 5B. Observations reported in Figures 5A,B led us to hypothesize that glomerular damage might result from mitochondria-derived ROS production. In order to test this, we measured mitochondrial superoxide anion production in isolated glomeruli using MitoSox fluorescent dye. Figure 5C shows that in the HS diet fed group, glomeruli had increased levels of MitoSox fluorescence intensity, indicative of differential mitochondrial ROS production between the NS and HS groups. Glomeruli isolated from rats fed a HS diet had elevated levels of superoxide production compared to NS, suggesting mitochondrial damage and/or their antioxidant system impairment, supporting the data described above. Interestingly, qPCR analysis (Figure 5D) revealed that in freshly isolated glomeruli the expression of *Gpx4* (encoding for glutathione peroxidase 4, which belongs to a family of proteins that catalyze the reduction of hydrogen peroxide, organic hydroperoxides, and lipid hydroperoxides, and protect cells against oxidative damage) was significantly reduced. We also reported the downregulation of the overall antioxidant system in the HS renal cortex (Figure 5E, measured with a Trolox-based assay), which is in line with data reported in Figures 5A,C,D.

## Discussion

The Dahl SS rat is a well-established and characterized rat model of SS hypertension, which displays renal lesions virtually identical to human hypertensive nephrosclerosis (Rapp, 1982; Rapp and Dene, 1985). We confirmed that rats obtained from Charles River recapitulated the phenotype typically observed after a HS diet challenge in this strain. We showed that these animals displayed an increase in systolic BP, kidney hypertrophy, and renal histological lesions (protein casts, glomerular scarring). This study focused on the role of renal cortical and particularly glomerular, mitochondria in the development of kidney injury, using a unique preparation of freshly isolated glomeruli.

It is widely accepted that in AKI and/or chronic kidney disease (CKD) such as DN, mitochondrial biogenesis is significantly affected, and thus could be one of the major reasons for renal tissue damage (Hall and Schuh, 2016; Bhargava and Schnellmann, 2017; Duann and Lin, 2017; Eirin et al., 2018). Surprisingly, little is known about mitochondrial function and biogenesis in SS hypertension. Here we demonstrate for the first time to our knowledge that while Dahl SS rats seem to have a largely preserved renal mitochondrial biogenesis, mitochondrial respiratory function declines in association with morphological changes in the mitochondrial glomeruli. We suggest this based on our data (Figure 2), which revealed very limited differences in mtDNA copy number, OXPHOS proteins, and PGC1α expression comparison between NS and HS diet fed groups. Consistent with this observation, we detected no differences in expression of *Atp5f1a*, *Cycs*, *Ppargc1a*, and *Cox4i1* genes in isolated glomeruli. Therefore, our data imply that the major mitochondrial changes in glomeruli during SS hypertension may be functional.

Indeed, the extracellular flux analyzer assay performed on freshly isolated glomeruli revealed dramatic differences in mitochondrial OCR, suggesting an impairment of glomerular mitochondrial function and inability to produce sufficient ATP to satisfy energy needs. We showed that the glomerular mitochondria from the HS group kidneys lost a significant portion of their reserve respiratory capacity. This is an important diagnostic parameter for mitochondrial bioenergetics, which can be indicative of mitochondrial stress, and help us determine how close to their functional limit the mitochondria are operating (Brand and Nicholls, 2011). In contrast to our bioenergetic data however, we did not detect any differences in Complex V activity between the groups. A possible explanation could be that while bioenergetic parameters were measured in intact glomeruli, Complex V activity was assessed in freeze–thawed glomerular pellets. In earlier studies, kidneys from Dahl SS rats were reported to display apoptosis related to mitochondrial release of cytochrome C and subsequent activation of caspase-9 and caspase-3 (Ying and Sanders, 2001). Zheleznova et al. (2012) reported that mTAL cells and mitochondria in the outer medulla of SS rats fed a HS diet (8% for 7 days) exhibited lower rates of oxygen utilization compared to those from the SS.13BN rats; the authors concluded that mTAL mitochondria energetics is changed in SS rats, which leads to a reduction in O_2_ utilization efficiency. This is in accordance with our data demonstrating decreased basal, ATP-linked and maximal respiration, as well as spare respiratory capacity in isolated glomeruli from Dahl SS rats fed a HS diet vs. NS.

In our experiments, increased oxidative stress and reduced antioxidant capacity were observed in renal glomeruli from SS rats fed a HS diet, which can be a contributing factor in glomerulosclerosis development under a HS challenge. Excessive mitochondrial H_2_O_2_ production has been shown previously in kidney disease, for instance, in mice fed a high fat diet (Ruggiero et al., 2011) or in the urine of mice fed a Western diet (Decleves et al., 2011). Our results are in accordance with a hypothesis that excessive H_2_O_2_ production and mitochondrial ROS leakage may be a common mechanism underlying pathogenesis in many forms of kidney disease, including SS hypertensive nephrosclerosis. Mitochondrial structural changes observed with EM potentially implicate the podocyte as a target cell in the glomerulus, however, further studies are required to draw definite conclusions. Salehpour et al. (2015) revealed that kidneys of Dahl SS rats have reduced levels of mitochondrial ETC activity and enhanced oxidative stress; specifically, when fed a HS diet, this strain exhibited significantly lower tissue redox ratios compared to the NS fed rats (Salehpour et al., 2015). We observed Complex V and IV level reduction in the renal cortex (but no apparent changes in Complex V activity or OXPHOS expression in isolated glomeruli), which partially agrees with the data by Ying and Sanders (2001) that showed inappropriate mitochondria release of Cytochrome C in the kidney tissue of Dahl SS rat. Interestingly, Lee et al. (2014) found increased mitochondrial OXPHOS in the renal proximal tubule cells of SHR rats compared to Wistar rats and identified the pyruvate dehydrogenase complex as a determinant of such increased mitochondrial metabolism. We believe that these discrepancies may be attributed to mechanistic dissimilarities in the origin and progression of hypertension in SHR and Dahl SS rats. A recent manuscript by Wang et al. (2017) established that renal mitochondria from Dahl SS rats fed HS diet (8%, 2 weeks) exhibit a lower activity of fumarase, isocitrate dehydrogenase, and succinyl-CoA synthase compared with SS.13BN salt-resistant control rats, as well as reduced ATP production, membrane potential, and SOD activity. Our data indicated that similar mitochondrial impairment manifested in Dahl SS rats when a lesser salt challenge was introduced (4%, 3 weeks). An earlier study by He et al. (2014) using TEM analysis showed that mitochondrial and ER ultrastructural abnormalities occurred in the medullary TALs of SS rats prior to the development of histological injury, potentially contributing to the subsequent development of metabolic and functional renal dysfunction (He et al., 2014). However, that study was focused on renal tubules; therefore, our data are the first to demonstrate the ultrastructural changes in the mitochondria from the podocytes in the Dahl SS rat fed a HS diet, including swelling, loss of cristae, and, in some cases, mitophagy.

**FIGURE 6:**
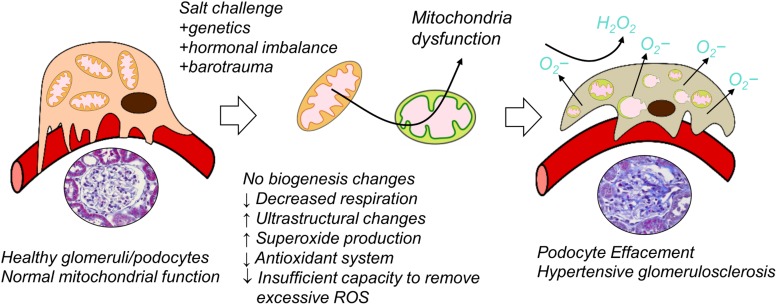
Suggested schematic illustration of the contribution of renal mitochondrial dysfunction to glomerular damage occurring in salt-sensitive (SS) hypertension.

To sum up, our study is the first one to report reduced oxygen consumption, impairments in bioenergetics, downregulation of renal antioxidant system, and increased oxidative stress specifically in the renal cortical glomeruli from Dahl SS rats fed a HS diet (Figure 6). We demonstrated that bioenergetic changes are not necessarily correlated with mitochondrial biogenesis in the renal cortex of the Dahl SS rats developing hypertensive nephrosclerosis. However, additional studies should be devoted to the factors inducing mitochondrial damage in the glomeruli, the timeline of damage development, and the use of these phenomena as a potential diagnostic tool in clinics (especially targeting renal mitochondria biogenesis vs. mitochondrial function in SS hypertension).

## Data Availability Statement

The datasets generated for this study are available on request to the corresponding author.

## Ethics Statement

The animal study was reviewed and approved by the MUSC Institutional Animal Care and Use Committee.

## Author Contributions

MD, IP, AN, VV, CK, RS, MF, GB, NS, A-LN, DI, and KS performed the experiments, and acquired, analyzed, and interpreted the data. DI, KS, and EM designed the study, interpreted and analyzed the data, and drafted the manuscript. All authors provided approval for the publication of the final manuscript.

## Conflict of Interest

The authors declare that the research was conducted in the absence of any commercial or financial relationships that could be construed as a potential conflict of interest.
